# Supplementary material for: Promiscuous enzymatic activity-aided multiple-pathway network design for metabolic flux rearrangement in hydroxytyrosol biosynthesis
Source: Nat Commun. 2019 Feb 27;10:960. doi: 10.1038/s41467-019-08781-2 (PMC6393456; doi:10.1038/s41467-019-08781-2)
Supplement: Supplementary file 1 — Supplementary Information [file 41467_2019_8781_MOESM1_ESM.pdf]

Promiscuous enzymatic activity-aided multiple-pathway network design for metabolic flux rearrangement in hydroxytyrosol biosynthesis

Chen *et al*.

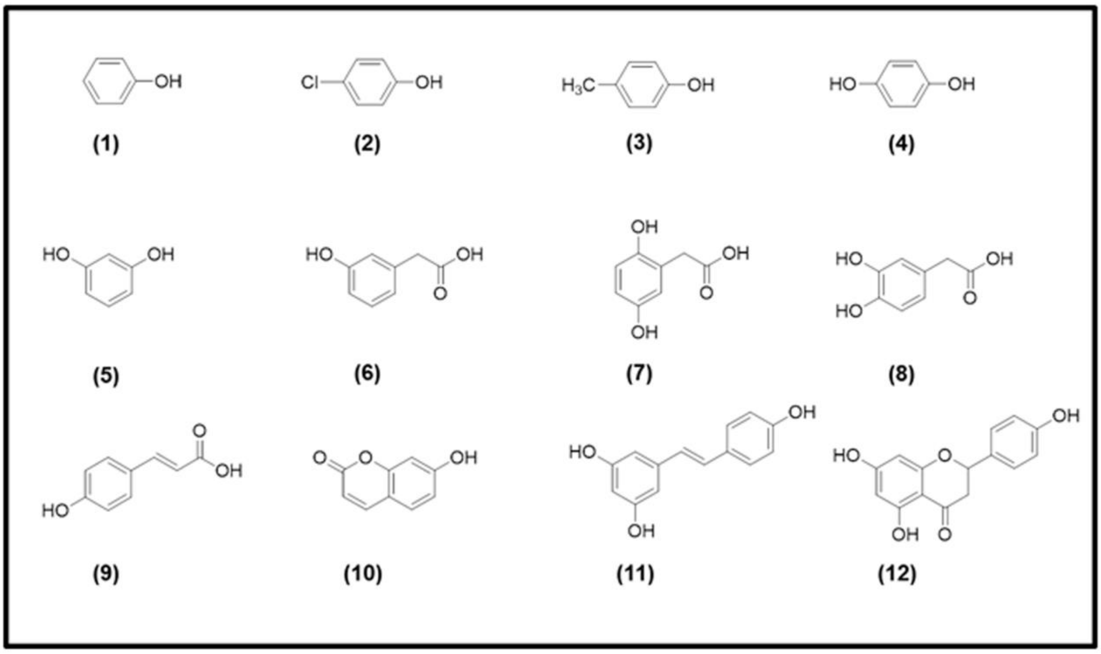

Supplementary Figure. 1. Reported substrates of wild-type HpaBC[1-2].

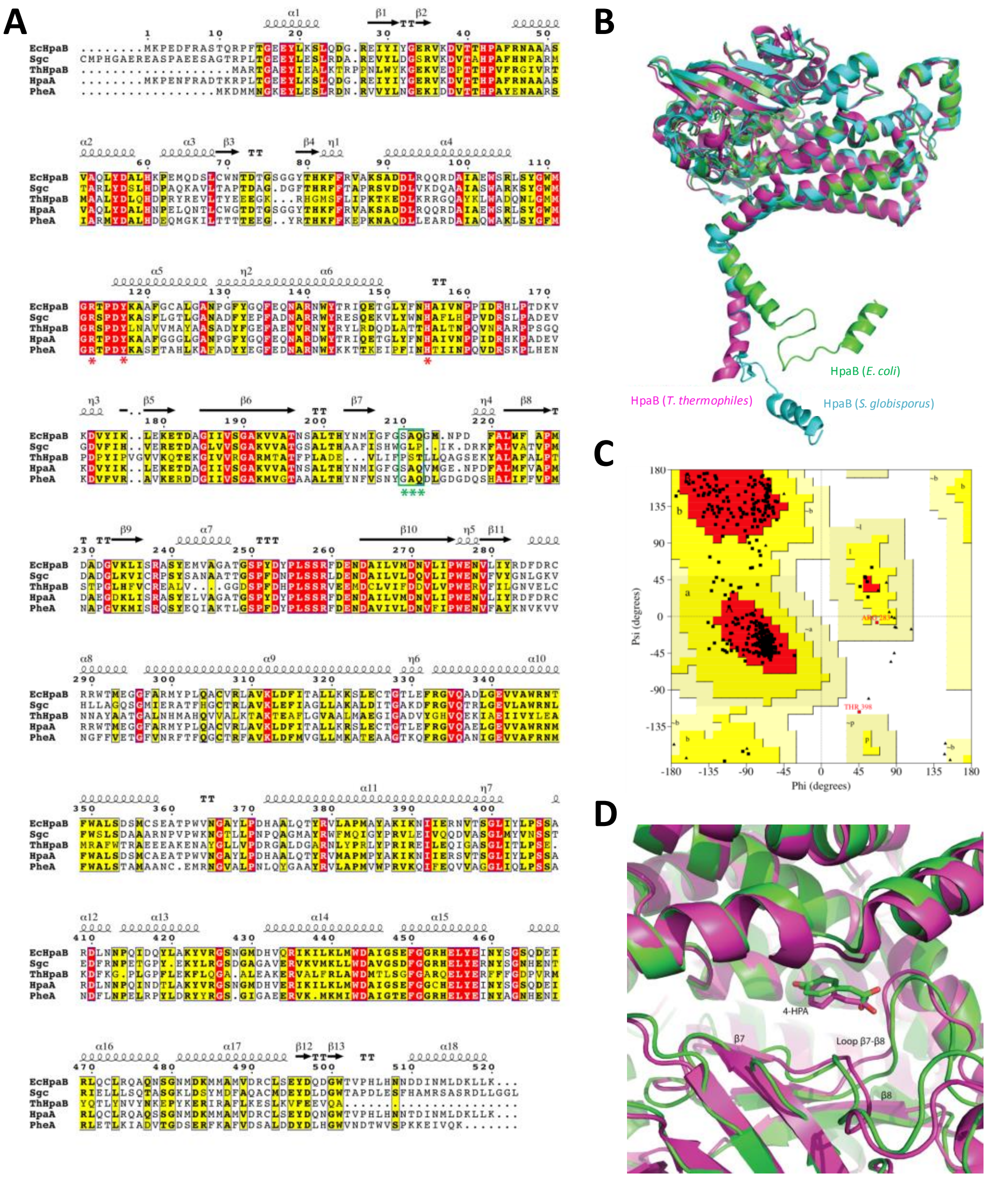

Supplementary Figure. 2. Modeling the structure of HpaB from *E. coli*. (A) Sequence alignment of HpaB from different species. Highly conserved residues were highlighted with red color, the catalytic residues R113, Y117 and H155 were marked with red markers. The residues involved substrate bindings were highlighted with green makers. (B) Alignment of *E. coli* HpaB model with HpaB structures from *Streptomyces globisporus* and *Thermus thermophiles*. The resulted RMSD were 0.605 and 1.048, for 4OO2[3] and 2YYJ[4], respectively. (C) The Ramachandran plot for HpaB model. (D) The substrate binding pocket comparison.

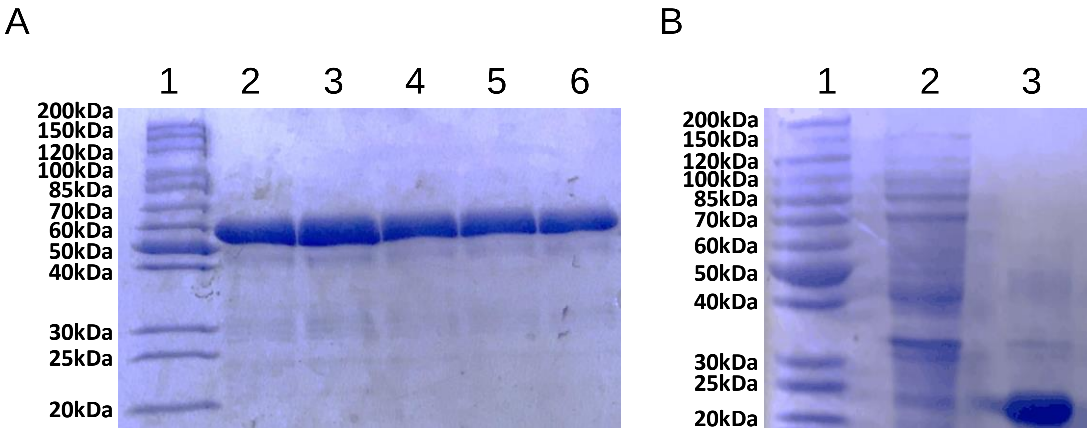

Supplementary Figure. 3. Protein expression and purification assessed with SDS-PAGE. (A) Purification of wild-type and mutant HpaBs. Lane 2 and 3 show all purified wild-type HpaB; Lane 4, 5 and 6 show purified HpaB muant A10, D11 and H7, respectively. (B) Purification of HpaC. Lane 2 shows the cell lysate of strain BL21(DE3) harboring plasmid pET28a; Lane 3 shows purified HpaC. Source data are provided in the Excel format Source Data file.

Table 1 Plasmids used in this study.

Plasmids	Description	Reference or source
Strains		
MC1061	For gene cloning	[5]
BL21(DE3)	For protein purification	Novagen
BW25113	For hydroxytyrosol biosynthesis	[6]
JW1380-KC	Strain from Keio collection in which *feaB* gene was relaced with FRT-flanked *kan* gene	[7]
BHYT	Strain BW25113 with *feaB* gene knocked out	This study
Plasmids		
pBAD18-Kan	Plasmid with pBR322 replication origin	[8]
pFA	Plasmid with p15A replication origin	This study
pRSF	Plasmid with pRSF3010 replication origin	This study
pFA-*hpaBC*	HpaBC expressed from plasmid pFA	This study
pFA-*tyo-tdc-hpaBC*(P1)	Pathway constructed in pFA	This study
pBAD18-*tyo-tdc-hpaBC*(P2)	Pathway constructed in pBAD18-Kan	This study
pRSF-*tyo-tdc-hpaBC*(P3)	Pathway constructed in pRSF	This study
pRSF-*tdc-tyo-hpaBC*(P4)	Pathway constructed in pRSF	This study
pRSF-*tyo-hpaBC-tdc*(P5)	Pathway constructed in pRSF	This study
pRSF-*hpaBC-tyo-tdc*(P6)	Pathway constructed in pRSF	This study
pRSF-*hpaBC-tdc-tyo*(P7)	Pathway constructed in pRSF	This study
pET28a	pET expression vector	Novagen
pET28a-*hpaB*	HpaB expressed from pET28a vector	This study
pET28a-*hpaC*	HpaC expressed from pET28a vector	This study

Table 2 Primers used in this study.

Primers	Sequence (5'-3')
pFA-for-*Xho*I	AAACTCGAGGATCTGGTACTAGTGGTGAATT
pFA-rev-*Nde*I	GGAATTCCATATGGGTTAATTCCTCCTGTTAGC
P1-gibson-1(for vector)	AAATGGAAGCTGCGATTTAAGATCTGGTACTAGTGGTGAA
P1-gibson-2(for vector)	ACCACATGCGGGTTGCTCATGGTTAATTCCTCCTGTTAGC
P1-gibson-3(for *tyo*)	GCTAACAGGAGGAATTAACCATGAGCAACCCGCATGTGGT
P1-gibson-4(for *tyo*)	TCATATTGTTTCTCCTTTACGCACGAATATCACGCA
P1-gibson-5(for *tdc*)	GTAAAGGAGAAACAATATGAAAAACGAAAAATTAGC
P1-gibson-6(for *tdc*)	TCATATTGTTTCTCCTTTATTTTACGTCGTAAATTT
P1-gibson-7(for *hpaBC*)	ATAAAGGAGAAACAATATGAAACCAGAAGATTTCCG
P1-gibson-8(for *hpaBC*)	TTCACCACTAGTACCAGATCTTAAATCGCAGCTTCCATTT
P2-gibson-1(for vector)	AAATGGAAGCTGCGATTTAAGATCTGGTACTAGTGGTGAA
P2-gibson-2(for vector)	ACCACATGCGGGTTGCTCATGGTTAATTCCTCCTGTTAGC
P2-gibson-3(for *tyo*)	GCTAACAGGAGGAATTAACCATGAGCAACCCGCATGTGGT
P2-gibson-4(for *tyo*)	TCATATTGTTTCTCCTTTACGCACGAATATCACGCA
P2-gibson-5(for *tdc*)	GTAAAGGAGAAACAATATGAAAAACGAAAAATTAGC
P2-gibson-6(for *tdc*)	TCATATTGTTTCTCCTTTATTTTACGTCGTAAATTT
P2-gibson-7(for *hpaBC*)	ATAAAGGAGAAACAATATGAAACCAGAAGATTTCCG
P2-gibson-8(for *hpaBC*)	TTCACCACTAGTACCAGATCTTAAATCGCAGCTTCCATTT
P3-gibson-1(for vector)	AAATGGAAGCTGCGATTTAAGATCTGGTACTAGTGGTGAA
P3-gibson-2(for vector)	ACCACATGCGGGTTGCTCATGGTTAATTCCTCCTGTTAGC
P3-gibson-3(for *tyo*)	GCTAACAGGAGGAATTAACCATGAGCAACCCGCATGTGGT
P3-gibson-4(for *tyo*)	TCATATTGTTTCTCCTTTACGCACGAATATCACGCA
P3-gibson-5(for *tdc*)	GTAAAGGAGAAACAATATGAAAAACGAAAAATTAGC
P3-gibson-6(for *tdc*)	TCATATTGTTTCTCCTTTATTTTACGTCGTAAATTT
P3-gibson-7(for *hpaBC*)	ATAAAGGAGAAACAATATGAAACCAGAAGATTTCCG
P3-gibson-8(for *hpaBC*)	TTCACCACTAGTACCAGATCTTAAATCGCAGCTTCCATTT
P4-gibson-1(for vector)	AAATGGAAGCTGCGATTTAAGATCTGGTACTAGTGGTGAA

P4-gibson-2(for vector)	GCTAATTTTTCGTTTTTCATGGTTAATTCCTCCTGTTAGC
P4-gibson-3(for *tdc*)	GCTAACAGGAGGAATTAACCATGAAAAACGAAAAATTAGC
P4-gibson-4(for *tdc*)	TCATATTGTTTCTCCTTTATTTTACGTCGTAAATTT
P4-gibson-5(for *tyo*)	ATAAAGGAGAAACAATATGAGCAACCCGCATGTGGT
P4-gibson-6(for *tyo*)	TCATATTGTTTCTCCTTTACGCACGAATATCACGCA
P4-gibson-7(for *hpaBC*)	GTAAAGGAGAAACAATATGAAACCAGAAGATTTCCG
P4-gibson-8(for *hpaBC*)	TTCACCACTAGTACCAGATCTTAAATCGCAGCTTCCATTT
P5-gibson-1(for vector)	TTCACCACTAGTACCAGATCTTATTTTACGTCGTAAATTT
P5-gibson-2(for vector)	ACCACATGCGGGTTGCTCATGGTTAATTCCTCCTGTTAGC
P5-gibson-3(for *tyo*)	GCTAACAGGAGGAATTAACCATGAGCAACCCGCATGTGGT
P5-gibson-4(for *tyo*)	TCATATTGTTTCTCCTTTACGCACGAATATCACGCA
P5-gibson-5(for *hpcBC*)	TCATATTGTTTCTCCTTTACGCACGAATATCACGCA
P5-gibson-6(for *hpcBC*)	TCATATTGTTTCTCCTTTAAATCGCAGCTTCCATTT
P5-gibson-7(for *tdc*)	TTAAAGGAGAAACAATATGAAAAACGAAAAATTAGC
P5-gibson-8(for *tdc*)	AAATTTACGACGTAAAATAAGATCTGGTACTAGTGGTGAA
P6-gibson-1(for vector)	AAATTTACGACGTAAAATAAGATCTGGTACTAGTGGTGAA
P6-gibson-2(for vector)	CGGAAATCTTCTGGTTTCATGGTTAATTCCTCCTGTTAGC
P6-gibson-3(for *hpcBC*)	GCTAACAGGAGGAATTAACCATGAAACCAGAAGATTTCCG
P6-gibson-4(for *hpcBC*)	TCATATTGTTTCTCCTTTAAATCGCAGCTTCCATTT
P6-gibson-5(for *tyo*)	TTAAAGGAGAAACAATATGAGCAACCCGCATGTGGT
P6-gibson-6(for *tyo*)	TCATATTGTTTCTCCTTTACGCACGAATATCACGCA
P6-gibson-7(for *tdc*)	GTAAAGGAGAAACAATATGAAAAACGAAAAATTAGC
P6-gibson-8(for *tdc*)	TTCACCACTAGTACCAGATCTTATTTTACGTCGTAAATTT
P7-gibson-1(for vector)	TGCGTGATATTCGTGCGTAAGATCTGGTACTAGTGGTGAA
P7-gibson-2(for vector)	CGGAAATCTTCTGGTTTCATGGTTAATTCCTCCTGTTAGC
P7-gibson-3(for *hpcBC*)	GCTAACAGGAGGAATTAACCATGAAACCAGAAGATTTCCG
P7-gibson-4(for *hpcBC*)	TCATATTGTTTCTCCTTTAAATCGCAGCTTCCATTT
P7-gibson-5(for *tdc*)	TTAAAGGAGAAACAATATGAAAAACGAAAAATTAGC
P7-gibson-6(for *tdc*)	TCATATTGTTTCTCCTTTATTTTACGTCGTAAATTT

P7-gibson-7(for *tyo*)	ATAAAGGAGAAACAATATGAGCAACCCGCATGTGGT
P7-gibson-8(for *tyo*)	TTCACCACTAGTACCAGATCTTACGCACGAATATCACGCA
hpaBC-Saturated-fwd	ATTGGCTTCGGCNNSNNSNNSGTGATGGGCGAA
hpaBC-Saturated-rev	TGACCCACGGCGTTGCTTCTGAACACATCGAGTCACTC
hpaB-for-*Nde*I	GGAATTCCATATGAAACCAGAAGATTTCCGCG
hpaB-rev-*Xho*I	AAACTCGAGTTATTTCAGCAGCTTATCCAGC
hpaC-for-*Nde*I	GGAATTCCATATGCAATTAGATGAACAACGC
hpaC-rev-*Xho*I	AAACTCGAGTTAAATCGCAGCTTCCATTT

Table 3 Sequence of the *tyo* gene after codon optimization.

Gene name	DNA sequence after codon optimization
tyo	ATGAGCAACCCGCATGTGGTGATTGTGGGTGCAGGTTTTGCAGGCC<br>TGGTGGCGGCGCGTGAACTGCAGATGGCCGGTGTGGATGTGGAAA<br>TTGTGGAAGCGCGTGATCGTGTGGGCGGCCGTGCATGGACCGAAG<br>AACGTATGGGTCGTCCGCTGGAACTGGGTGCAACCTGGGTGCATTG<br>GATGCAGCCGCATGTGTGGAGCGAAATTACCCGTTATGATCAGAGC<br>ATTTATCCGAGCCCGTTTTGCGATGATGCGTATTGGATTACCGGCGG<br>CCGTGTGGAACATGGTACCGAAGCAGATCTGGATGCAGCACTGGCA<br>CGTCCGATGGCGAAAATTTTTGAAGATAGCCGTGAATTTTTCCCGTA<br>TCCGTATGAACCGCTGCATGTGCTGGATGAAAGCAGCGGCAGCACC<br>CCGGAACTGCGTGAACGTTTTCGTGCGGCGGATCAGGGCAGCGTG<br>CTGGATTGCCTGAAAGGCGGCGATTTTACCCAGGAAGAACGTGATC<br>TGTGCGATGCGTATTGGAGCGCGGCGTATATTGGCGATCCGCATCA<br>GGGCAGCCCGCTGATGGCGAAACAGTGGGCGGCGCTGAGCGATCA<br>TCGTCTGAGCCTGGTGGATGAACAGACCCTGCGTTTTAAACTGACC<br>CATGGCATGCGTGGCCTGTATGAAAACATTGCGGCGGATCTGCGTT<br>GCCCGATTCGTCTGAACACCCCGGTGACCGCAGTGGATCATCGTAG<br>CGATGGTGCAACCGTGACCCTGGGTACCGGTGAAAAAATTAGCTGC<br>GATAGCGTGATTGTGACCGTGCCGGTGGGTGCACTGCCGACCATTG<br>AATTTACCCCGGGTCTGCCGAGCGGTATGCGTACCGTGATTGATCA<br>GCGTTGGAACAGCACCGGCTGCAAAATTTGGGTGAAAGTGAAAGG<br>CCATCATAGCATTCTGGGCTATGCGCCGACCCCGCATAAAGCGGCG<br>GTGTTTCGTAGCGAATTTTTCATGGATGATGATACCACCATTTGCGTG<br>GGCTTTGGCAGCCATCATGATGCGGTGGATCTGACCGATCCGCGTG<br>ATGCGCAGGCGATTGTGGATCAGTGGCGTCCGGATCTGGAAGTGG<br>TGGATTGCACCGGTCATGATTGGGTGGCAGATCGTTGGAGCGGTCA<br>GGCATGGGCAACCCTGCGTAGCGGCCAGTTTACCAACGGCTGGCA<br>TCATTTTCGTAGCACCGATAGCCGTCTGCGTTTTGCAGGTGCAGATT<br>GGGCGCGTGGCTGGCGTGGCGTGGTGGTGGATGGTGCAATTGAAA<br>CCGGTCTGAGCACCGCGCGTGATGTGCTGCGTGATATTCGTGCGTA<br>A

Supplementary References:

1. Lin, Y. & Yan, Y. Biotechnological production of plant-specific hydroxylated phenylpropanoids. *Biotechnol. Bioeng.* **111**, 1895-1899 (2014).
2. Prieto, M.A., Perez-Aranda, A. & Garcia, J.L. Characterization of an *Escherichia coli* aromatic hydroxylase with a broad substrate range. *J. Bacteriol.* **175**, 2162-2167 (1993).
3. Chang, C.Y., *et al.* Crystal structures of SgcE6 and SgcC, the two-component monooxygenase that catalyzes hydroxylation of a carrier protein-tethered substrate during the biosynthesis of the enediyne antitumor antibiotic C-1027 in *Streptomyces globisporus*. *Biochemistry-US* **55**, 5142-5154 (2016).
4. Kim, S.H., *et al.* Crystal structure of the oxygenase component (HpaB) of the 4-hydroxyphenylacetate 3-monooxygenase from *Thermus thermophilus* HB8. *J. Biol. Chem.* **282**, 33107-33117 (2007).
5. Casadaban, M.J. & Cohen, S.N. Analysis of gene control signals by DNA fusion and cloning in *Escherichia coli*. *J. Mol. Biol.* **138**, 179-207 (1980).
6. Datsenko, K.A. & Wanner, B.L. One-step inactivation of chromosomal genes in *Escherichia coli* K-12 using PCR products. *Proc. Natl. Acad. Sci. USA* **97**, 6640-6645 (2000).
7. Baba, T. & Mori, H. The construction of systematic in-frame, single-gene knockout mutant collection in *Escherichia coli* K-12. *Methods Mol. Biol.* **416**, 171-181 (2008).
8. Guzman, L.M., Belin, D., Carson, M.J. & Beckwith, J. Tight regulation, modulation, and high-level expression by vectors containing the arabinose P_{BAD} promoter. *J. Bacteriol.* **177**, 4121-4130 (1995).
